# Identification of Major Degradation Products of Ketoconazole

**DOI:** 10.3797/scipharm.1107-18

**Published:** 2011-10-13

**Authors:** Rajendra A. Mhaske, Shirish Sahasrabudhe

**Affiliations:** 1 Sharon Bio Medicine, 312, C-Wing, BSEL Tech Park, Opp. Vashi Railway Station, Sector 30 (A), Vashi, Navi-Mumbai-400703, India; 2 Shri Jagdishprasad Jhabarmal Tiberewala University, J. B. Nagar, Andheri (E), Mumbai–400059, India

**Keywords:** Ketoconazole, Stress degradation, Hydrolysis degradent, Oxidative degradent, LC-MS

## Abstract

Analytical methods were developed for the identification of major degradation products of Ketoconazole, an antifungal agent. The stressed degradation of Ketoconazole drug substance was performed under acid, base, thermal, photo and oxidative stress conditions. The major degradation was observed under acid, base and oxidative stress conditions. The degradation study was performed on Inertsil ODS-3V, length 100 X diameter 4.6 mm, particle size 3 μm column using gradient method. These degradants were identified by LC-MS technique.

## Introduction

Ketoconazole is an antifungal drug approved by the US FDA in 1981. Only a few analytical methods for the determination of the drug in biological samples and in the presence of other drugs have been reported [1–12]. The photodegradation behavior of Ketoconazole has been reported by Staub et al [13]. The drug substance is official in Ph. Eur. but the specified impurities are not mentioned. The present study deals with understanding the degradation behavior of Ketoconazole by subjecting it to acid, base, aqueous, thermal, photo and oxidative stress conditions. Furthermore, the two major degradation impurities observed under stressed condition were identified by LC-MS techniques, elemental analysis, NMR, and their structures were justified through mechanistic explanation.

## Experimental

### Material and reagents

Ketoconazole drug substance was obtained from Sharon Biomedichem (Navi Mumbai, India). All the chemicals and reagents, hydrochloric acid, sodium hydroxide, hydrogen peroxide (30 %), tetrabutylammonium hydrogen sulphate, acetonitrile and methanol were used of analytical grade, while a millipore milli Q plus water purification system (Milford, USA) was used to prepare distilled water (>18 μΩ).

### Instruments

Integrated HPLC system, Ultimate 3000 manufactured by Dionex (Germany) was used for method development and method validation. This system consisted of a quaternary gradient pump, auto sampler, column oven and a photodiode array detector. PC installed Chromeleon software was used to record and to integrate the chromatograms. The analysis was carried out at ambient temperature. LCMS system, Agilent ion trap 6310 was used for mass fragmentation analysis. NMR experiments were recorded on Bruker 500 Mz spectrometer. Photostability studies were performed in a photostability chamber from Thermolab (India).

### Chromatographic conditions

#### Analytical HPLC conditions

Inertsil ODS (Length: 100 mm, Diameter: 4.6 mm, Particle size: 3 μm) analytical column was used as a stationary phase. The flow rate was 2.0 ml min^−1^ and the detector was set at 220 nm. The volume of the sample solution injected was 10 μl. The gradient mobile phase consisted of Mobile phase A {(Acetonitrile: 3.4 g/l solution of tetrabutylammonium hydrogen sulphate (5:95 V/V)}: and Mobile phase B {(Acetonitrile: 3.4 g/l solution of tetra-butylammonium hydrogen sulphate (50:50 V/V)}. A membrane filter of 0.45 μm porosity was used to filter and degas the mobile phase. (Gradient program as mentioned in Tab. 1).

#### Analytical LC–MS conditions

Inertsil ODS (Length: 100 mm, Diameter: 4.6 mm, Particle size: 3 μm) analytical column was used as a stationary phase. The flow rate was 2.0 ml min^−1^ and the detector was set at 220 nm. The volume of the sample solution injected was 10 μL. The gradient mobile phase consisted of Mobile phase A (Water) and Mobile phase B (Acetonitrile). A membrane filter of 0.45 μm porosity was used to filter and degas the mobile phase. The gradient program as mentioned in Table 2. The LC-Mass condition was set using Nebulizer 50 PSI, dry gas temperature 350 degree and source ESI positive.

### Stress degradation of drug substance

Stress studies were carried out under acid, base, thermal, photo and oxidative stress conditions.

#### Acid Hydrolysis

250.0 mg of test sample + 2ml 1N HCl into 25 ml volumetric flask. Sample heated on boiling water bath at 100 deg, withdrawn at 2 min and 8 min, respectively, then neutralized with 1N NaOH solution and make up the volume to 25 ml with methanol. Pipette out 4 ml into 50 ml volumetric flask and dilute to volume with methanol.

**Fig. 1:**
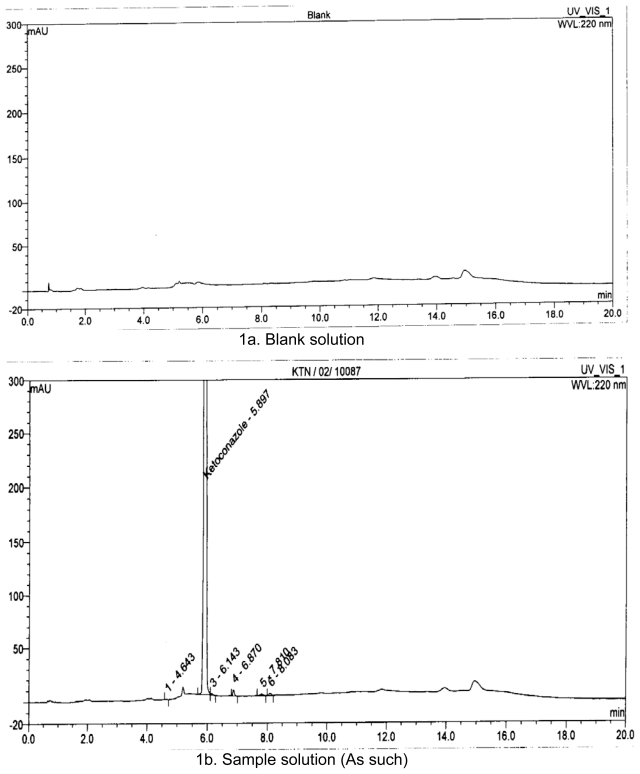

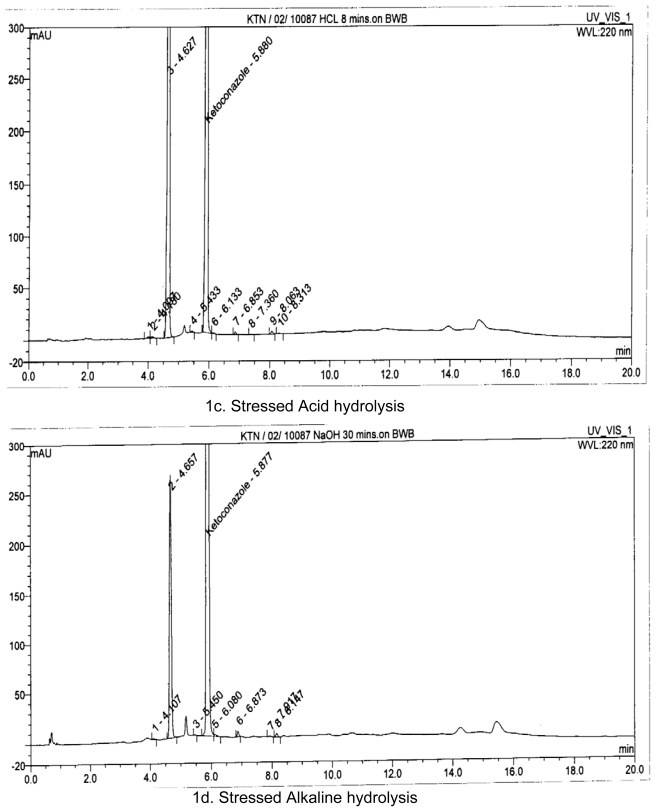

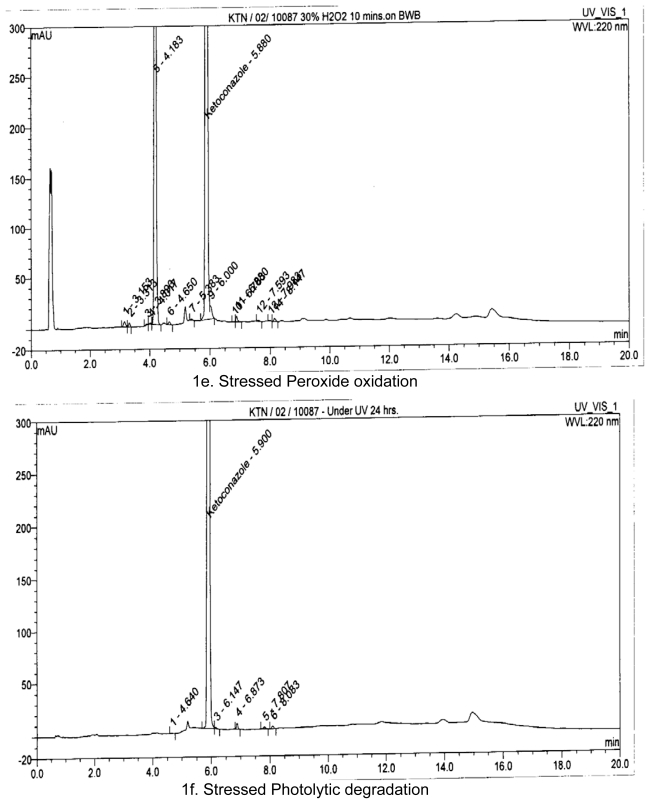

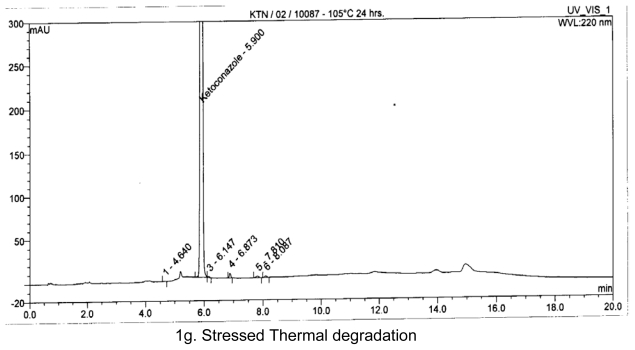
HPLC Chromatograms for Stressed conditions

One unknown degradation impurity was observed under acidic condition (Table 3 and figure 1c). In figure 1c, the main degradation product is unknown impurity at RRT 0.80.

#### Base Hydrolysis

250.0 mg of test sample + 2ml 1N NaOH into 25 ml volumetric flask. Sample heated on boiling water bath for 10 min and 30 min, respectively, then neutralized with 1N HCl solution and make up the volume to 25 ml with methanol. Pipette out 4 ml into 50 ml volumetric flask and dilute to volume with methanol.

One unknown degradation impurity was observed under basic condition which is the same as observed under acidic condition (Table 4 and figure 1d). In figure 1d, the main degradation product is unknown impurity at 0.80.

#### Oxidation

250.0 mg of test sample + 2ml 30%H2O2 into 25 ml volumetric flask and heated for 10 min on boiling water bath. Make up the volume to 25 ml with methanol. Pipette out 4 ml into 50 ml volumetric flask and dilute to volume with methanol.

One unknown degradation impurity was observed under oxidative stress condition and it is different from the impurity observed under acidic/basic condition (Table 5 and figure 1e). In figure 1.e, the main degradation product is unknown impurity at RRT 0.72.

#### Thermal

Test sample of Ketoconazole was subjected to thermal degradation by exposure to oven at 105°C for 24h and 60°C at 5 days and 10 days. 250.0 mg test sample of Ketoconazole were dissolved and diluted with methanol to 25 ml. Pipette out 4 ml into 50 ml volumetric flask and dilute to volume with methanol.

#### Photolysis

About 250.0 mg test sample of Ketoconazole is kept for UV degradation for 24hours at 254 nm wavelength and then dissolved and diluted with methanol to 25 ml. Pipette out 4 ml into 50 ml volumetric flask and dilute to volume with methanol. The drug substance was found stable under photo and thermal stress conditions as shown in below (Table 6, figure 1f and 1g).

## Preparation of Impurities

**Fig. 2:**
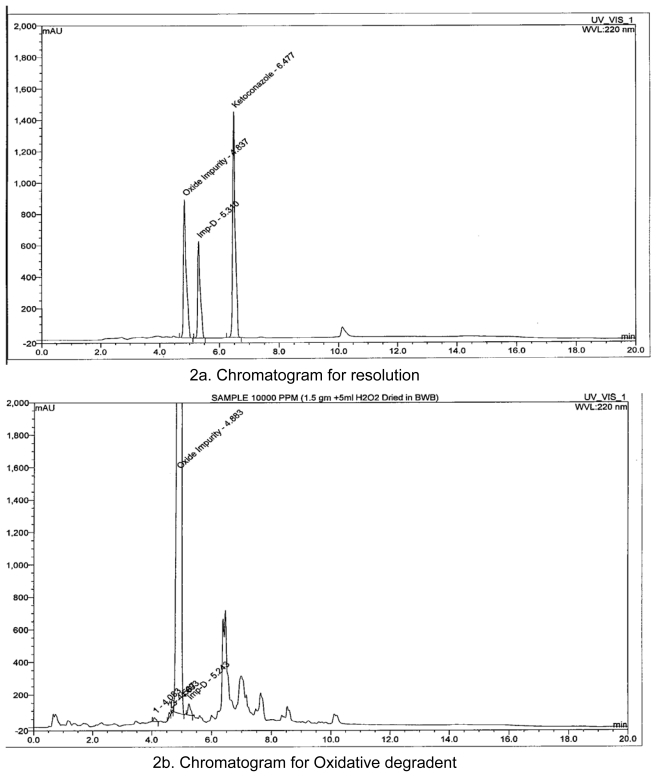
HPLC Chromatograms for preparation Oxidative degradent

**Fig. 3:**
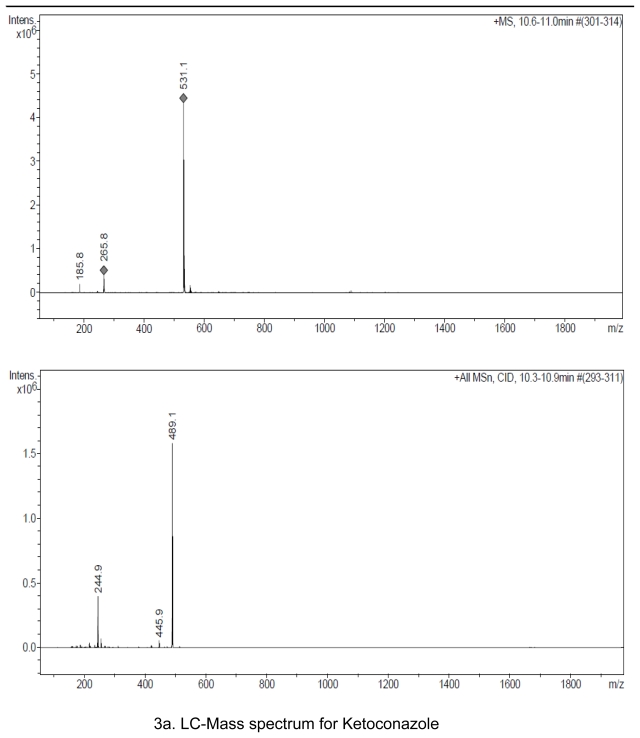

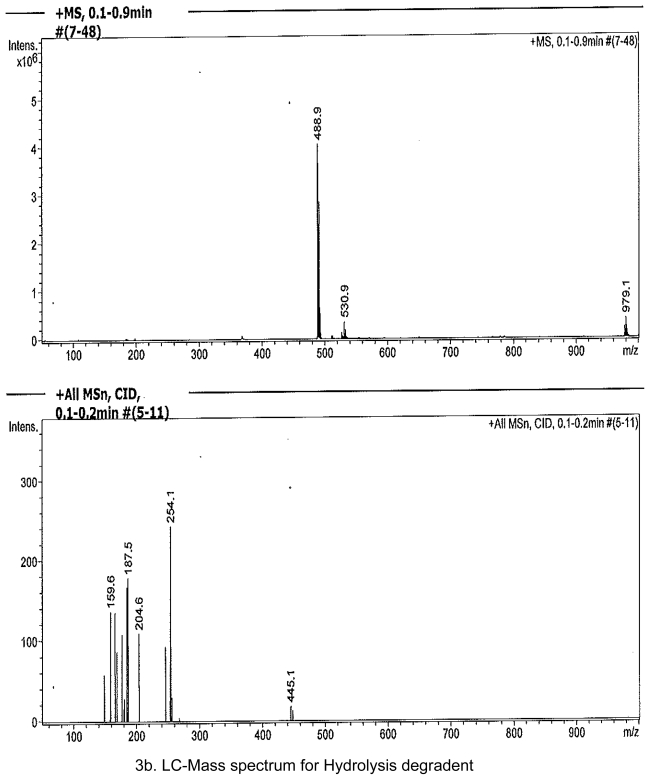

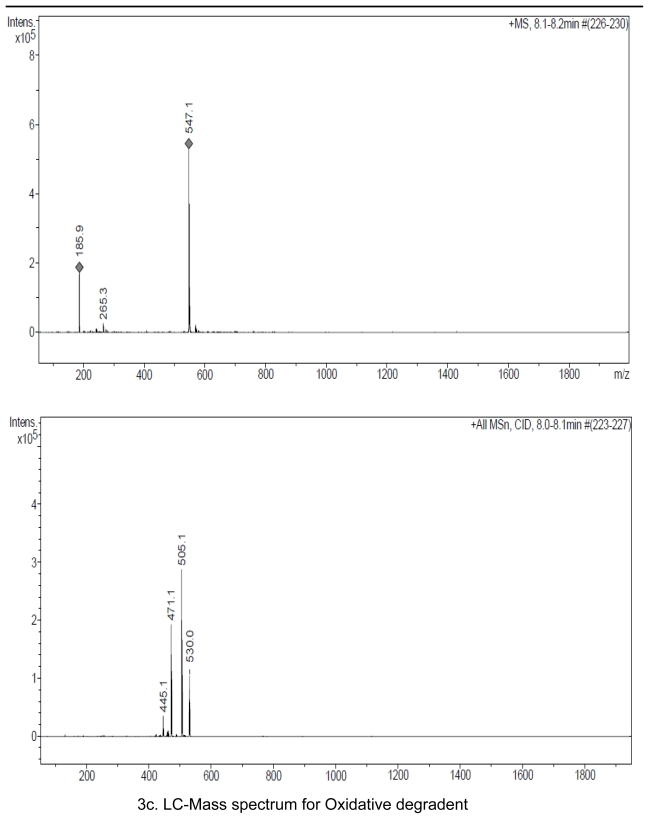
LC-Mass spectrum for Ketoconazole and degradent products

Hydrolysis degradent, Impurity D as per Ph. Eur., is synthesized in-house and identified by HPLC analysis, Mass spectrometer (figure 3b) and elemental analysis (figure 6b). Oxidative degradent is prepared in-house by degradation of Ketoconazole with 30% hydrogen peroxide by heating up to evaporate to dryness at 80**°**C. Ketoconazole gets converted to its N-oxide and identified by HPLC (figure 2b), LCMS analysis (figure 3c), NMR analysis (figure 5b) and Elemental analysis (figure 6c, 6d).

## Elemental analysis

Elemental analysis (CHNO) of Ketoconazole, Hydrolysis degradent and Oxidative degradent performed and results shown below Table 7 and 8.

## NMR analysis

NMR analysis of Ketoconazole and the oxidative degradent were performed and the results are shown in Table 9.

## Results and discussion

**Fig. 4:**
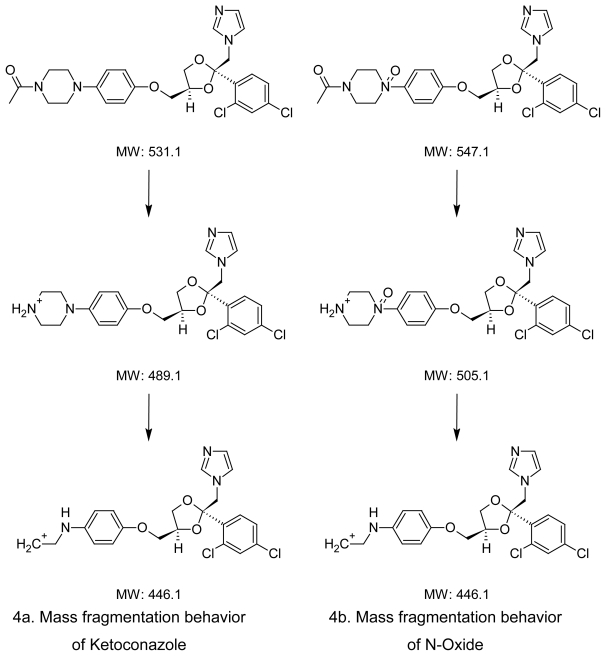
Mass fragmentation behavior of Ketoconazole and N-Oxide

**Fig. 5:**
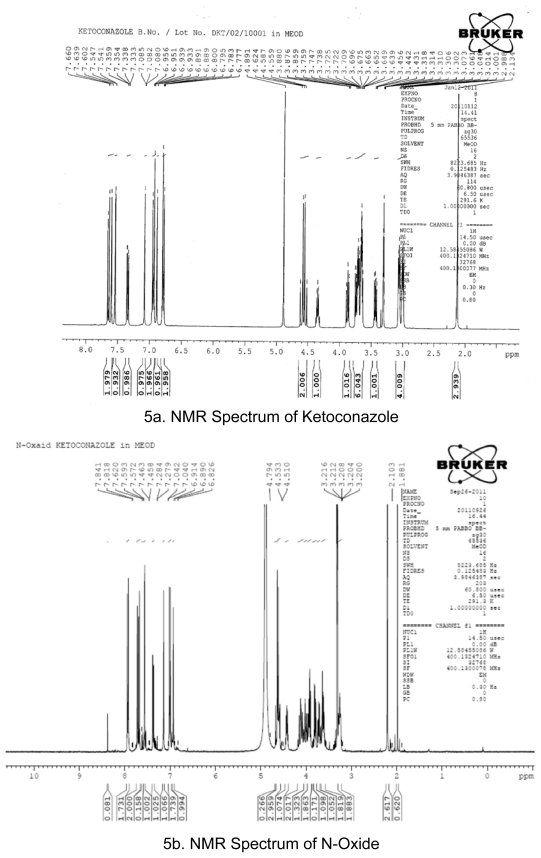
NMR Spectrum of Ketoconazole and N-Oxide

**Fig. 6:**
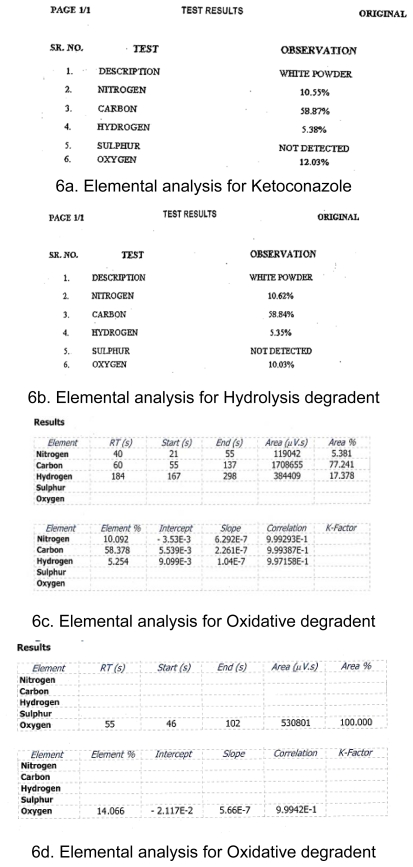
Elemental analysis for Ketoconazole and degradent products

The degradation of Ketoconazole was performed under different stress conditions. Two major degradants are observed under stress degradation. One is hydrolysis product of Ketoconazole observed under acid/base condition and the other one is oxidative degradent observed under oxidative stress condition. The identification of oxidative degradent was achieved by LC-MS, NMR and Elemental analysis. The LC-MS data shows the mass 547.43 amu which exactly increase in the mass 16 amu from the Ketoconazole drug substance having mass 531.43 amu, which indicate the formation of N-oxide. LC-MS spectrums and fragmentation behavior of N-Oxide are given in figure 3c and 4b. Also, the elemental analysis of N-oxide shows the increase in oxygen atom (figure 6d), while in the case of hydrolysis decrease in oxygen atom compare to Ketoconazole. The NMR analysis of oxidative degradent shows the shifting of protons signal from their original position in Ketoconazole due to introduction electronegative oxygen atom (figure 5b).

Hence, the formation of the oxidative degradation product from the drug as shown below is only due to the N-oxide formation at the piperazine ring. The lone pair at the nitrogen of the piperazine ring is more prone for oxidation to form an N-oxide. However, out of two nitrogen atoms, the electron pair on the nitrogen attached to the carbonyl group is participating in resonance delocalization with this group. Hence, the most possible N-Oxide at the nitrogen is at the one attached to the phenolic group (scheme 1).

Similar types of N-oxide degradents have been reported in the literature [14–16].

## Conclusions

The Stress degradation on Ketoconazole was carried out under different acid, base, thermal, photo and oxidative stress conditions. The drug was found susceptible to acid, base and oxidative stress degradation. The unknown degradation products formed in the oxidative and hydrolysis stressed sample were identified using LC–MS and elemental analysis (CHNO). The investigations of oxidative and hydrolysis degradent will help to take proper care during selection of excipients in formulation, storage, packaging and handling of the drug product.

## Figures and Tables

**Sch. 1:**
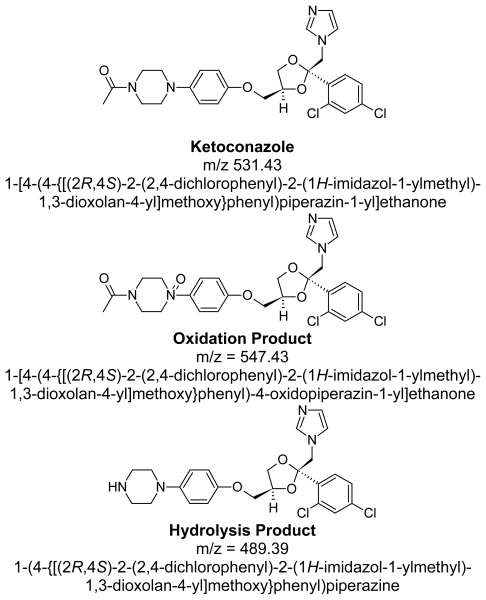
Structure of elucidated compounds

**Tab. 1:** Mobile Phase gradient for HPLC chromatographic method

Time (min)	Mobile phase A (% V/V)	Mobile phase B (& V/V)
0	100	0
10	0	100
15	0	100
17	100	0
20	100	0

**Tab. 2:** Mobile Phase gradient for LC-MS method

Time (min)	Water (% V/V)	Acetonitrile (% V/V)
0	100	0
20	0	100
30	0	100

**Tab. 3:** Results of Acid degradation (1M HCl)

Name of compounds	RRT	Sample “as such”	Initial	4 hours at 25°C	2 minutes heating at 100°C	8 minutes heating at 100°C
Unknown	~0.66	ND	ND	0.052	ND	0.085
Unknown	~0.72	ND	ND	0.105	0.042	0.080
Unknown	~0.76	ND	ND	ND	0.036	ND
Unknown	~0.80	0.005	0.018	0.878	5.778	22.122
Ketoconazole	~1.00	99.535	99.510	98.213	91.417	69.620
Unknown	~1.03	0.049	0.054	0.050	0.085	0.046
Unknown	~1.09	ND	ND	ND	0.045	ND
Unknown	~1.11	ND	ND	0.017	0.046	0.085
Unknown	~1.19	0.196	0.200	0.206	0.220	0.110
Unknown	~1.32	0.104	0.102	0.009	0.139	ND
Unknown	~1.38	0.114	0.110	0.106	0.126	ND

**Tab. 4:** Results of Base degradation (1M NaOH)

Name of compounds	RRT	Sample “as such”	Initial	4 hours at 25°C	10 minutes heating at 100°C	30 minutes heating at 100°C
Unknown	~0.66	ND	0.083	0.124	0.088	ND
Unknown	~0.72	ND	0.091	0.137	0.090	0.023
Unknown	~0.80	0.005	0.010	0.439	5.328	10.702
Ketoconazole	~1.00	99.535	99.310	98.055	92.172	88.821
Unknown	~1.03	0.049	0.076	0.136	0.079	0.109
Unknown	~1.09	ND	ND	ND	ND	ND
Unknown	~1.11	ND	0.014	0.004	0.161	0.163
Unknown	~1.19	0.196	0.200	0.212	0.100	0.037
Unknown	~1.32	0.104	0.099	0.114	0.094	0.130
Unknown	~1.38	0.114	0.111	0.120	ND	ND

**Tab. 5:** Results of Oxidative degradation (30% H_2_O_2_)

Name of compounds	RRT	Sample “as such”	Initial	4 hours at 25°C	10 minutes heating at 100°C
Unknown	~0.54	ND	ND	ND	0.219
Unknown	~0.56	ND	ND	ND	0.134
Unknown	~0.66	ND	0.125	0.149	0.084
Unknown	~0.72	ND	0.172	0.860	23.528
Unknown	~0.80	0.005	0.007	0.008	0.078
Ketoconazole	~1.00	99.535	99.185	98.478	74.995
Unknown	~1.03	0.049	0.124	0.116	0.500
Unknown	~1.19	0.196	0.213	0.212	0.174
Unknown	~1.32	0.104	0.012	0.010	0.047
Unknown	~1.38	0.114	0.124	0.124	0.122

**Tab. 6:** Results of Thermal and UV degradation

Name of compound	RRT	Sample “as such”	105°C at 24 hours	60°C at 5 days	60°C at 10 days	24 hours at 254 nm
Unknown	~0.80	0.005	0.008	0.008	0.007	0.007
Ketoconazole	~1.00	99.535	99.576	99.578	99.561	99.536
Unknown	~1.03	0.049	0.046	0.050	0.055	0.053
Unknown	~1.19	0.196	0.179	0.183	0.190	0.195
Unknown	~1.32	0.104	0.103	0.093	0.102	0.097
Unknown	~1.38	0.114	0.085	0.084	0.083	0.109

**Tab. 7:** Results of Elemental analysis

	Ketoconazole MW 531.43		Hydrolysis degradent MW 488.9		Oxidative degradent MW 547.43	

Element	% Calc.	% Found	% Calc.	% Found	% Calc.	% Found
C	58.71	58.87	58.91	58.84	56.994	58.378
H	5.27	5.38	5.32	5.35	5.115	5.254
N	10.54	10.55	11.45	10.62	10.230	10.092
O	12.04	12.03	9.82	10.03	14.614	14.066

**Tab. 8:** No of atoms present in molecule

Element	Ketoconazole	Hydrolysis degradent	Oxidative degradent
C	26	24	26
H	28	26	28
N	4	4	4
O	4	3	5
Observed Molecular formula	C_26_H_28_Cl_2_N_4_O_4_	C_24_H_26_Cl_2_N_4_O_3_	C_26_H_28_Cl_2_N_4_O_5_

**Tab. 9:** Results of NMR analysis

	Ketoconazole		Oxidative degradent	
	
	1H δ (ppm)	No of protons	1H δ (ppm)	No of protons
1 CH_3_ group	2.134	2.939	2.103, 1.881	3.237
7 CH_2_ and 1 CH group	2.988–3.897	15.075	3.200–4.794	15.525
Aromatic protons	6.777–7.660	9.757	6.826–7.841	9.796
